# Processing nanoporous organic polymers in liquid amines

**DOI:** 10.3762/bjnano.10.179

**Published:** 2019-09-09

**Authors:** Jeehye Byun, Damien Thirion, Cafer T Yavuz

**Affiliations:** 1 Water Cycle Research Center, Korea Institute of Science and Technology (KIST), 5 Hwarang-ro 14, Seoul, 02792, Korea; 2 Graduate School of Energy, Environment, Water and Sustainability (EEWS), Korea Advanced Institute of Science and Technology (KAIST), 291 Daehak-ro, Yuseong-gu, Daejeon, 34141, Korea; 3 Department of Chemical and Biomolecular Engineering, Korea Advanced Institute of Science and Technology (KAIST), 291 Daehak-ro, Yuseong-gu, Daejeon, 34141, Korea

**Keywords:** film, liquid amine, nanoparticles, nanoporous polymer, processibility

## Abstract

Rigid network structures of nanoporous organic polymers provide high porosity, which is beneficial for applications such as gas sorption, gas separation, heterogeneous (photo)catalysis, sensing, and (opto)electronics. However, the network structures are practically insoluble. Thus, the processing of nanoporous polymers into nanoparticles or films remains challenging. Herein, we report that nanoporous polymers made via a Knoevenagel-like condensation can be easily processed into nanoparticles (115.7 ± 40.8 nm) or a flawless film by using liquid amines as a solvent at elevated temperatures. FTIR spectra revealed that the carboxyl groups in the nanoporous polymers act as reactive sites for amines, forming new functionalities and spacing the polymeric chains to be dissolved in the liquid amines. The processed film was found to be CO_2_-philic despite the low surface area, and further able to be transformed into a fine carbon film by thermal treatment.

## Introduction

Nanoporous polymers offer permanent porosity along with robust and light-weight frameworks. The building-block approach for nanoporous polymers allows for a nearly infinite variety of architectures by changing chemistry and geometry of the monomers [1]. New discoveries on nanoporous polymers have been made based on different combinations of monomers yielding unique properties to the polymer structures. In particular, the high porosity of nanoporous polymers has gained significant attention in various applications such as gas adsorption and storage [2], water purification [3], energy storage [4], and catalysis [5], where the large and permanent voids in nanoporous polymers provide room to accommodate target substrates and guest molecules. A series of nanoporous polymers have been reported, namely covalent organic frameworks [6], conjugated microporous polymers [7], covalent triazine frameworks [8], porous aromatic frameworks [9], porous polymer networks [10], and polymers of intrinsic microporosity [11]. We have been working on nanoporous covalent organic polymers (COP), focusing on structural durability and economic sustainability for gas capture [12] and water treatment applications [13].

Nanoporous polymers are classified as network polymers, and such network polymers do not dissolve in common organic solvents but rather swell. Despite the advantages of network structures, the nanoporous polymers can be hardly processed into the desired morphologies such as small particulates, films, and membranes because of the insolubility. So far, the generation of nanoparticles and films made out of nanoporous polymers have been mostly done through initial synthetic controls such as miniemulsion polymerization [14], ionic complexation [15], and electrochemical polymerization [16], which require sophisticated techniques. There are only a few reports that show the processing of nanoporous polymers into the desired morphologies, for instance, the nanoporous polymers were directly processed into nanoparticles with different sizes [17]. However, extremely harsh conditions (piranha solution, 160 °C) were necessary to break the polymeric chains, indicating that the processing of nanoporous polymers is still challenging. Obviously, more applications were possible, if the nanoporous polymers could be fabricated into nanoparticles and films.

In this work, nanoporous polymers built via Knoevenagel-like condensations are made to be soluble in liquid amines and show structural processibility. At elevated temperatures (ca. 100 °C), the condensate nanoporous polymers were completely dissolved in neat liquid amines in a few seconds, and the resulting polymer solution was able to be transformed into either nanoparticles or flawless films. This new feature of processibility was underpinned by FTIR, in which the formation of amide groups between the carbonyl units on nanoporous polymers and the alkylamines was responsible for the dissolution of polymers. The obtained polymer film exhibited high CO_2_-philicity by having nitrogen-rich surfaces. The film could be also turned into a fine carbon film by heat treatment in inert atmosphere.

## Results and Discussion

### Processing COP-100 in liquid amines

**Figure 1:**
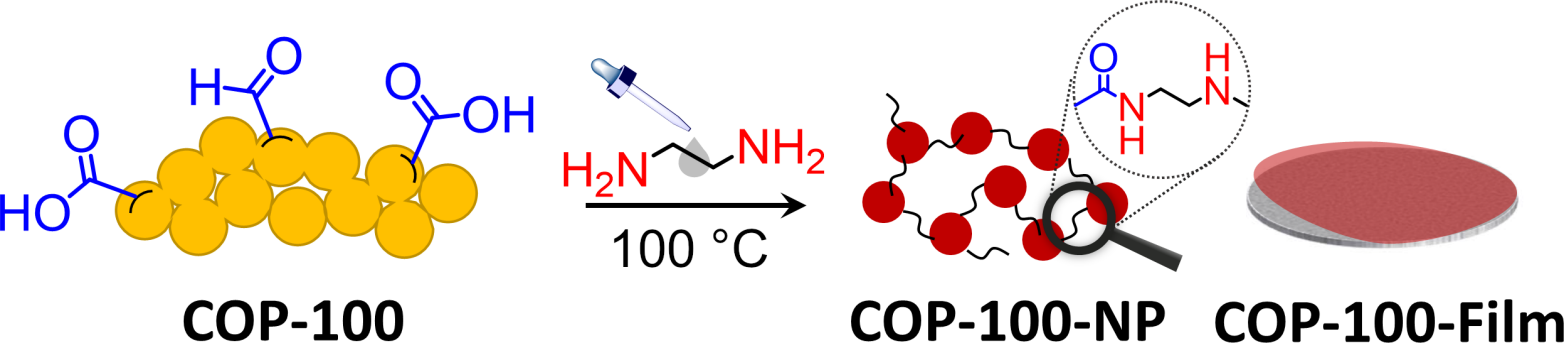
Schematic illustration of processible COP-100 in EDA to form either nanoparticles (COP-100-NP) or films (COP-100-Film) at 100 °C. The terminal groups of carboxylic acids and unreacted aldehydes are responsible for interparticle connections.

COP-100 is a network polymer that can be synthesized in one step from 1,3,5-benzenetriacetonitrile and terephthaldehyde via Knoevenagel-like condensation [18] (synthesis details can be found in Supporting Information File 1). The reaction proceeded smoothly at room temperature in the presence of potassium *tert*-butoxide with short reaction time (less than 3 h) and almost quantitative yield (>95%). A typical Knoevenagel reaction is known to form fully conjugated structures by two steps, where the nucleophilic addition of carbonyl takes place on the deprotonated α-CN position, further followed by the deprotonation of second α-CN proton and the successive dehydration [19–20]. COP-100, however, was intended to have a hydroxylated structure by using a bulky base (*t*-BuOK) and an aprotic solvent (THF), where the second deprotonation and the concomitant dehydration does not occur as the bulky base cannot easily access inside the polymer network. From our previous findings, the hydroxylated COP-100 exhibited the highest surface area of 82.3 m^2^·g^−1^ among all the tested combinations of base and solvent [18]. The mild reaction conditions of COP-100 further left carbonyl units in the structure to be reactive sites for further processing. These reactive sites could be unreacted aldehyde or carboxylic acid generated by disproportionation of aldehyde units [21], which can quickly react with amines to form imine or amide bonds, respectively. When ethylenediamine (EDA) was added to COP-100 under elevated temperatures, the short amine chains penetrated into COP-100 and formed the new functionalities with carbonyl units, creating room between the polymeric backbones to be dissolved in liquid amines. The amine-induced solubility of COP-100 facilitated the formation of small nanoparticles or polymeric film layers to give COP-100-NP and COP-100-Film, respectively (Figure 1).

**Figure 2:**
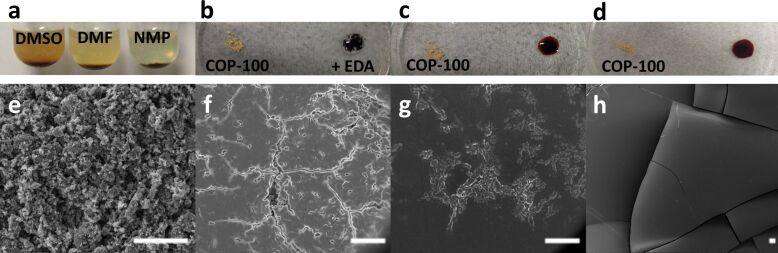
(a) Low solubility of COP-100 in polar aprotic solvents. (b) Instant color change of COP-100 by the addition of EDA dropwise at r.t., (c) dissolution of COP-100 in EDA at 100 °C in a few seconds, and (d) formation of COP-100 film at 100 °C in a few minutes (less than 5 min). SEM image of (e) pristine COP-100 powder, and EDA-treated COP-100 at 100 °C for (f) 1 min, (g) 3 min, and (h) 5 min (scale bar = 10 μm).

COP-100 is sparingly soluble in common polar aprotic solvents including DMF, DMSO, DMAc, and NMP (Figure 2a). Intriguingly, when COP-100 was treated with EDA, there was an instant color change from light yellow to deep red (Figure 2b). Under thermal treatment at 100 °C, the whole powder dissolved in the amine within a few seconds, forming a red solution (Figure 2c). The solution was further turned into a film within a few minutes under elevated temperatures (Figure 2d). The polymer solution in EDA was highly polar, and it was miscible with common polar solvents but not with non-polar solvents. The COP-100-Film, on the other hand, was not soluble in any solvents despite its brittle nature.

COP-100 in EDA was monitored using microscopic tools to understand its rapid phase change. As shown in Figure 2e, the original COP-100 consists of large polymeric granules aggregated with each other, and the size of each granule ranged from 100 to 300 nm. A TEM image of the COP-100 solution in EDA treated for a few seconds at 100 °C (Supporting Information File 1, Figure S1) shows that COP-100 granules were torn apart and separated from each other. In addition, the granules were covered with organic layers, presumably excess EDA. The solution was transferred on a silicon wafer to analyze the morphology changes by SEM as function of the heating time. When treated at high temperature for a short period of time (ca. 1 min), the granules were combined in a heap to form a cracked film surface (Figure 2f). The boundary layers between the granules were not demarcated as the heat treatment went on for 3 min (Figure 2g). After 5 min, as all the pieces were combined and covered with excess EDA, the surface became very clean, leading to a flawless film (Figure 2h). Since the film itself was thin and brittle, it was easily cracked, but the film did not show any polymeric granules exposed on the surface. The dissolution of polymer chains in a solvent is known to involve two transport processes; (i) solvent diffusion and (ii) chain disentanglement [22]. When the COP-100 was reacted with EDA, there was a color change of the COP, indicating the formation of an infiltration layer of EDA on the polymeric surface. As the treatment time progressed, further penetration of the EDA made the surface layer swollen so that bulk polymer cracked into smaller units that can behave like liquids.

**Figure 3:**
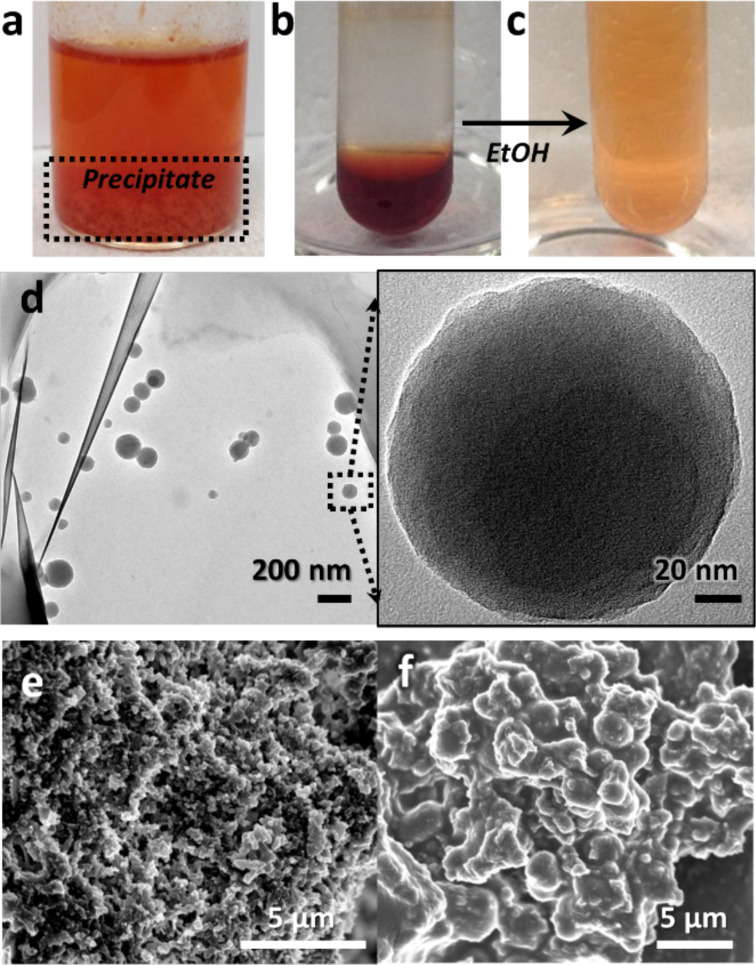
Effect of amine equivalence in solubilizing COP-100. (a) COP-100 dissolved in 8 equivalent EDA which precipitated by the addition of ethanol, (b) COP-100 dissolved in 50 equivalent EDA, and (c) diluted solution of (b) in ethanol. (d) TEM image of COP-100-NP obtained from the solution (b). SEM image of COP-100 precipitates after the addition of (e) 5 equivalent EDA and (f) 8 equivalent EDA.

During processing COP-100, the amount of EDA varied according to the molar ratio between the nitrogen content of COP-100 and the molecular weight of EDA. Interestingly, even a tiny amount of EDA can dissolve COP-100, showing significant solubility COP-100 in liquid amines. The amount of EDA, however, mattered when the further process went on. For instance, when only a little amount of EDA was added to COP-100, the mixture became a red polymeric solution, but the COP-100 polymer precipitated (COP-100-Precip.) if a polar solvent, i.e., ethanol, was added (Figure 3a). It may be attributed that the amount of EDA was not enough to react with all COP-100. Thus, the unreacted COP-100, which is technically insoluble in any solvent, precipitated. When the amount of EDA was in large excess, the COP-100-Film became partially soluble in polar solvents. In order to make a stable polymer solution that does not form a polymer precipitate after the addition of polar solvents, the molar ratio between EDA and nitrogen content of COP-100 needs to be at least 10:1. At the same time, the critical molar ratio for the formation of an insoluble film in polar solvents was thus about 50:1 or less for EDA/COP-100.

A typical COP-100 solution with 50 equiv EDA, showing a clear red color (Figure 3b), could be easily mixed and diluted with ethanol (Figure 3c). When the diluted polymer solution was analyzed with TEM, separated polymer nanoparticles were observed showing an average size of 115.7 ± 40.8 nm (Figure 3d). The spherical shape of the polymer nanoparticles resulted from the split COP-100 granules covered with EDA. COP-100 precipitates with a lower amount of EDA, on the other hand, exhibited the aggregated form similarly to the original COP-100 (Figure 3e). After the addition of 8 equiv EDA, the COP-100 granules were fully covered with EDA layers, which is in the middle of a transformation into a film (Figure 3f).

### Possible mechanism of the processibility

**Figure 4:**
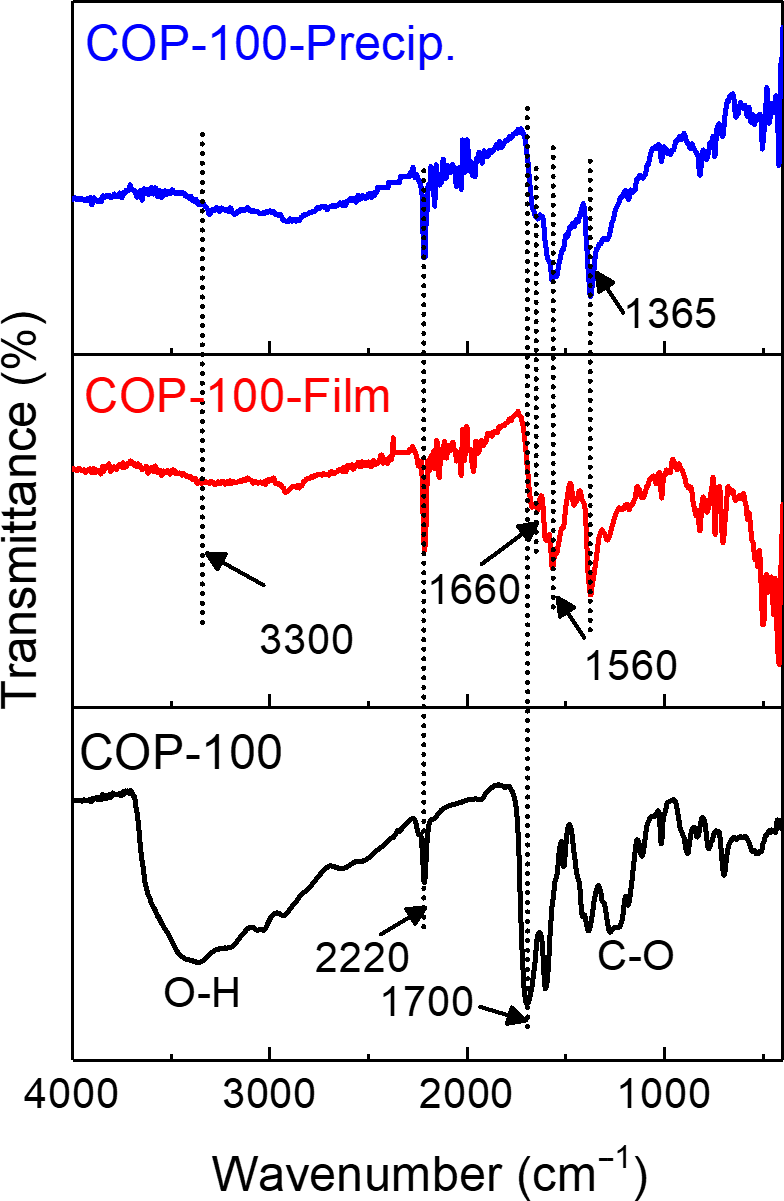
FTIR spectra of COP-100, COP-100-Film, and COP-100-Precip. COP-100 exhibited two peaks at 3400 cm^−1^ and 1375 cm^−1^ of O–H and C–O stretching vibrations of the hydroxylated structure, respectively.

The instant color change of COP-100 with EDA indicated a chemical reaction between COP-100 and EDA. Our initial hypothesis was the addition of EDA on the nitrile groups by a nucleophilic attack [23], so that the alkylamine chain connected to the nitrile functionality can increase the polarity of the polymeric chains, making COP-100 soluble. However, as shown in Figure 4, FTIR spectra of COP-100, COP-100-Film, and COP-100-Precip. showed no drastic change in the nitrile region (2200 cm^−1^). Moreover, the addition of an amine on a nitrile group cannot be achieved in a few seconds without catalyst despite the high temperature. The existence of carbonyl units at around 1700 cm^−1^ in COP-100 drew our attention. The carbonyl groups could originate either from unreacted terephthaldehyde or from the carboxylic acid generated during the work-up process with acid through the disproportionation of two adjacent aldehydes [21]. Aldehyde groups are known to generate imine bonds by the reaction with amines, however, a fast imine formation is most likely when an acid catalyst is present [24]. Alternatively, carboxylic acid could react with amines to form amide linkages at high temperatures [25], which corresponds to new peaks shown on the FTIR spectra of both COP-100-Film and COP-100-Precip. at 3300 cm^−1^, 1660 cm^−1^, and 1560 cm^−1^ for amide N–H, C=O, and C–N–H stretching vibrations, respectively [26]. The strong peak at 1365 cm^−1^ in both COP-100-Film and COP-100-Precip. spectra were attributed to the ethylene C–H stretching vibration of EDA. The direct formation of the amide functionality could be possibly due to the easy condensation with the hydrophobic nanoporous polymer at high temperature [27]. The COP-100-Film was formed much slower when the temperature was below 80 °C, supporting that the amide formation by the condensation reaction was crucial for processing the nanoporous polymer. The quick amide formation made the COP-100 granules torn apart through the interpenetration of EDA molecules into the polymeric chains. Consequently, as the EDA layers covered the COP-100 particles, a film could be generated with a clean surface.

**Figure 5:**
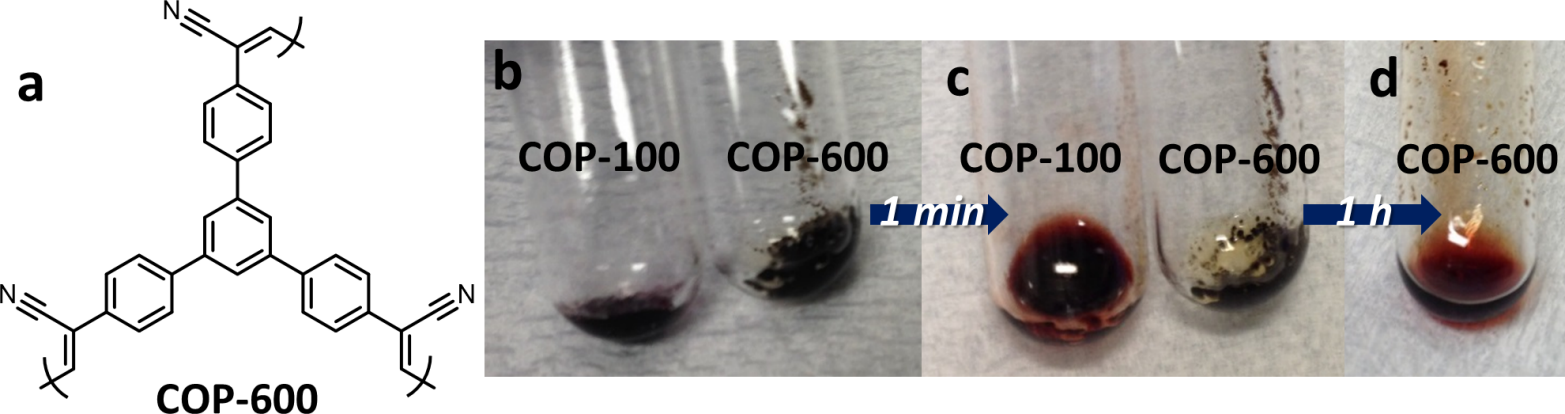
(a) Structure of COP-600 synthesized by Knoevenagel-like condensation. Solubility of COP-600 in EDA compared to COP-100; (b) addition of EDA at r.t., (c) heat treatment at 100 °C for 1 min and (d) for 1 h.

The amine-induced solubility was not restricted to only COP-100. COP-600 (Figure 5a), produced by the Knoevenagel-like condensation of 1,3,5-benzenetriacetonitrile and 1,3,5-tris(4-formylphenyl)benzene, was also solubilized in EDA at elevated temperatures. COP-600 was not soluble in organic solvents even at high temperatures but it was in the presence of EDA. COP-600 has an almost pore-free structure, thus the dissolution in the liquid amine took much longer compared to COP-100 due to the slower penetration of EDA. The rigid backbone of COP-600 with the triphenylbenzene core also contributed to the slower dissolution of polymer in EDA. As shown in Figure 5b, a red polymer solution of COP-600 could be obtained after 1 h of thermal treatment at 100 °C. The type of liquid amines could also be varied for solubilizing polymer structures (Supporting Information File 1, Figure S2). Among a series of common liquid amines, alkylamines, i.e., benzylamine and triethylenetetramine (TETA), were able to solubilize COP-100 at 100 °C. Allylamine, despite the amine structure, has a low boiling point (ca. 53 °C), which was not suitable for the high-temperature processing. Tertiary amines, i.e., triethylamine, did not solubilize COP-100 under the given conditions, confirming that the formation of the amide group was responsible for the polymer dissolution. The two control experiments with COP-600 and different liquid amines implied that nanoporous polymers made by Knoevenagel(-like) condensations can be easily processed in alkylamines, showing the wide applicability of the amine-induced processing method in terms of the structural variety of nanoporous polymers and amines.

### Properties of COP-100-Film

**Figure 6:**
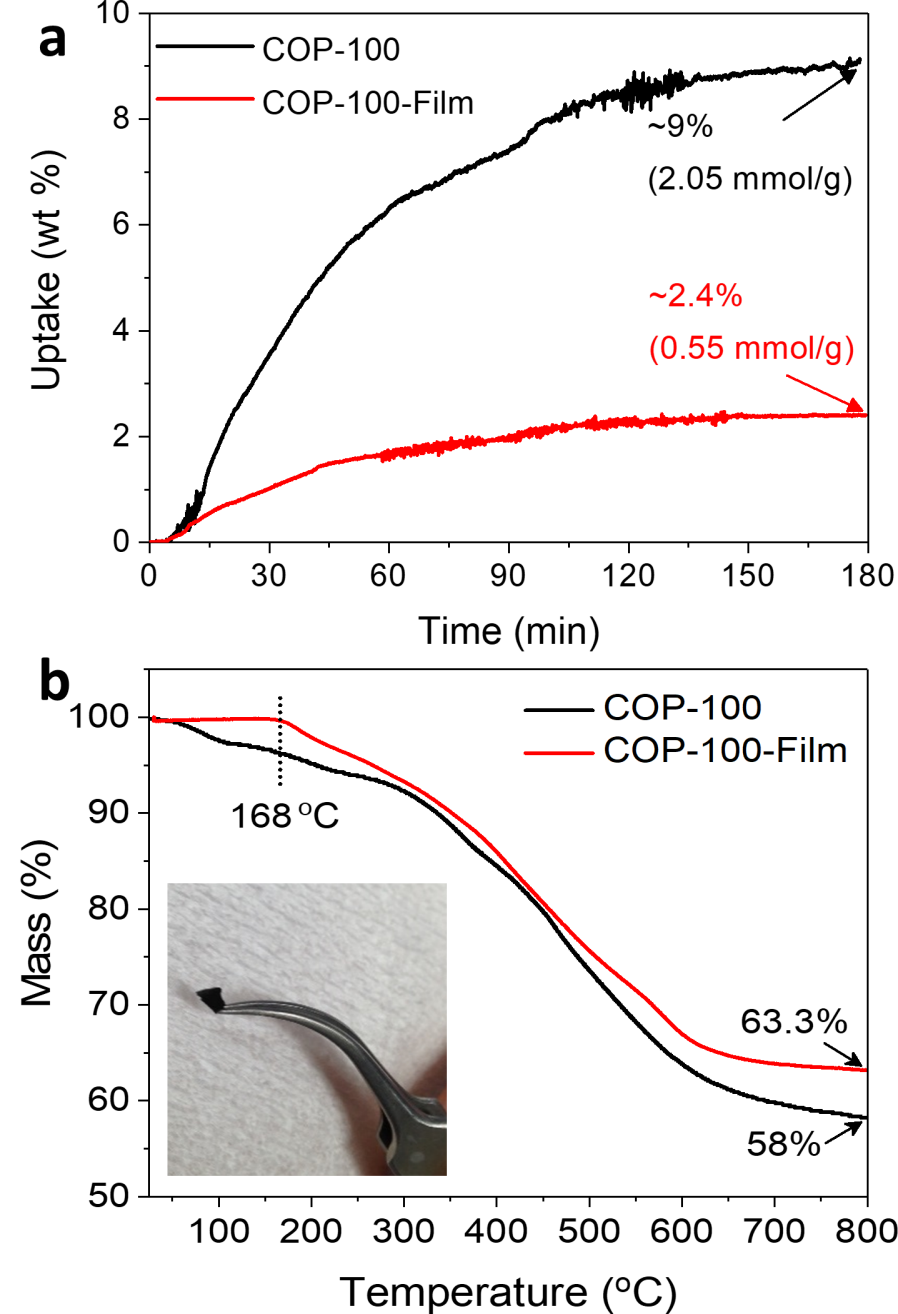
(a) Gravimetric CO_2_ adsorption isotherm of COP-100 and COP-100-Film at 40 °C. (b) Thermogravimetric analysis of COP-100 and COP-100-Film under N_2_ atmosphere. Inset displays a photograph of the carbonized COP-100-Film.

Once COP-100-Film formed, it did not dissolve in any common organic solvents as long as the amount of EDA was within the threshold range, from 10 to 50 equiv of COP-100. The amount of nitrogen in COP-100-Film varied as the EDA amount changed. Typically, after addition of 10 equiv EDA, the COP-100-Film exhibited a nitrogen content of 20% by elemental analysis, indicating about an increase of nitrogen content by about 76% compared to the original COP-100. The COP-100-Film with higher nitrogen content might be eligible for CO_2_ adsorption [28]. In a customized gravimetric analysis setup (Supporting Information File 1, Figure S3), COP-100 and COP-100-Film were tested for CO_2_ uptake, where 9% and 2.4% of CO_2_ sorption were observed in 3 h at 40 °C, respectively (Figure 6a). Despite the far worse CO_2_ adsorption performance compared to COP-100, COP-100-Film exhibited a moderate CO_2_ capture behavior even with the non-porous structure, owing to the nitrogen-rich surface.

Thermogravimetric analysis of COP-100-Film (Figure 6b) showed that there was an initial mass loss at around 165 °C, possibly due to the evaporation of unassociated EDA residue incorporated within the film. The final mass of the film at 800 °C was about 63%, which corresponds to the 5% mass gain compared to that of COP-100 at 800 °C (58%), indicating that the actual amount of EDA reacted with COP-100 was about 5% in mass. The film treated at 800 °C under inert conditions maintained its original shape, and the rigid texture of the annealed COP-100-Film indicated its high potential for fabricating a flat carbon surface on a variety of substrates (Figure 6b, inset) [29].

## Conclusion

In this study, a new phenomenon is discovered to nanoporous polymer networks. The nanoporous covalent organic polymer (COP-100) was dissolved in liquid amines via simple heat treatment, and the polymer solution could be processed into either nanoparticles or films. FTIR revealed that the carbonyl groups in the polymers were reactive sites that helped to solubilize the structures in liquid amines by forming amide functionality. The control experiment with COP-600 further confirmed that amine-induced processing method can be widely applied to nanoporous network polymers having carbonyl units, especially for structures produced via a Knoevenagel-like condensation. Future research will be focused on the in-depth characterization of porosity and chemical functionalities of the processed polymers. Nanosized porous polymers would yield a better surface area, and size control would be of interest for applications in which size matters such as heterogeneous catalysis [30]. The films and membranes processed from nanoporous polymers should be tested for gas/liquid adsorption applications [31], as the functionalities of nanoporous polymers remain intact even after the processing. The remaining amines and newly formed amide functionalities in the nanoporous polymers would provide an additional advantage for favorable interaction toward target sorbate molecules. A control in thickness and composition of the film should also be achieved when varying the type and the concentration of nanoporous polymers, which will affect the permeability and selectivity of polymer films for adsorption applications.

## Supporting Information

Synthetic details and methods. A TEM image and a photograph of dissolved polymers in liquid amines. A schematic illustration of gravimetric gas adsorption set-up.

File 1 Additional experimental details.

## References

[1] Wu D, Xu F, Sun B, Fu R, He H, Matyjaszewski K (2012). Chem Rev.

[2] Zhu Y, Long H, Zhang W (2013). Chem Mater.

[3] Alsbaiee A, Smith B J, Xiao L, Ling Y, Helbling D E, Dichtel W R (2016). Nature.

[4] Thomas A, Kuhn P, Weber J, Titirici M-M, Antonietti M (2009). Macromol Rapid Commun.

[5] Kaur P, Hupp J T, Nguyen S T (2011). ACS Catal.

[6] Côté A P, Benin A I, Ockwig N W, O'Keeffe M, Matzger A J, Yaghi O M (2005). Science.

[7] Xu Y, Jin S, Xu H, Nagai A, Jiang D (2013). Chem Soc Rev.

[8] Kuhn P, Antonietti M, Thomas A (2008). Angew Chem, Int Ed.

[9] Ben T, Ren H, Ma S, Cao D, Lan J, Jing X, Wang W, Xu J, Deng F, Simmons J M (2009). Angew Chem, Int Ed.

[10] Lu W, Yuan D, Zhao D, Schilling C I, Plietzsch O, Muller T, Bräse S, Guenther J, Blümel J, Krishna R (2010). Chem Mater.

[11] McKeown N B, Budd P M (2006). Chem Soc Rev.

[12] Patel H A, Karadas F, Canlier A, Park J, Deniz E, Jung Y, Atilhan M, Yavuz C T (2012). J Mater Chem.

[13] Byun J, Patel H A, Thirion D, Yavuz C T (2016). Nat Commun.

[14] Ma B C, Ghasimi S, Landfester K, Zhang K A I (2016). J Mater Chem B.

[15] Zhao Q, Dunlop J W C, Qiu X, Huang F, Zhang Z, Heyda J, Dzubiella J, Antonietti M, Yuan J (2014). Nat Commun.

[16] Gu C, Huang N, Gao J, Xu F, Xu Y, Jiang D (2014). Angew Chem, Int Ed.

[17] Zhu Y, Qiao M, Peng W, Li Y, Zhang G, Zhang F, Li Y, Fan X (2017). J Mater Chem A.

[18] Özdemir E, Thirion D, Yavuz C T (2015). RSC Adv.

[19] Bi S, Lan Z-A, Paasch S, Zhang W, He Y, Zhang C, Liu F, Wu D, Zhuang X, Brunner E (2017). Adv Funct Mater.

[20] Thirion D, Lee J S, Özdemir E, Yavuz C T (2016). Beilstein J Org Chem.

[21] Chung S-K (1982). J Chem Soc, Chem Commun.

[22] Rao J P, Geckeler K E (2011). Prog Polym Sci.

[23] Wang J, Xu F, Cai T, Shen Q (2008). Org Lett.

[24] Ciaccia M, Di Stefano S (2015). Org Biomol Chem.

[25] Rahman M, Kundu D, Hajra A, Majee A (2010). Tetrahedron Lett.

[26] Larkin P J (2018). Infrared and Raman Spectroscopy: Principles and Spectral Interpretation.

[27] Ojeda-Porras A, Hernández-Santana A, Gamba-Sánchez D (2015). Green Chem.

[28] Byun J, Je S-H, Patel H A, Coskun A, Yavuz C T (2014). J Mater Chem A.

[29] Yeo K, Kim J, Kim J (2018). J Nanosci Nanotechnol.

[30] Bond G C (1985). Surf Sci.

[31] Budd P M, Msayib K J, Tattershall C E, Ghanem B S, Reynolds K J, McKeown N B, Fritsch D (2005). J Membr Sci.

