# Supplementary material for: Processing nanoporous organic polymers in liquid amines
Source: Beilstein J Nanotechnol. 2019 Sep 9;10:1844–50. doi: 10.3762/bjnano.10.179 (PMC6753686; doi:10.3762/bjnano.10.179)
Supplement: File 1 — Additional experimental details. [file Beilstein_J_Nanotechnol-10-1844-s001.pdf]

BEILSTEIN JOURNAL OF NANOTECHNOLOGY

Supporting Information

for

Processing nanoporous organic polymers in liquid amines

Jeehye Byun, Damien Thirion and Cafer T. Yavuz

Beilstein J. Nanotechnol. **2019,** *10,* 1844–1850. doi:10.3762/bjnano.10.179

Additional experimental details

License and Terms: This is a supporting information file under the terms of the Creative Commons Attribution License (http://creativecommons.org/licenses/by/4.0). Please note that the reuse, redistribution and reproduction in particular requires that the authors and source are credited.
The license is subject to the *Beilstein Journal of Nanotechnology* terms and conditions: (https://www.beilstein-journals.org/bjnano)

Synthesis of COP-100

The synthetic details and characteristics of COP-100 can be found in our previous report [1]. In a typical experiment, 2,2',2''-(benzene-1,3,5-triyl)triacetonitrile (0.45 g, 2.31 mmol) and terephthaldehyde (0.47 g, 3.5 mmol) were dissolved in dry THF (40 mL) under inert conditions. Potassium *tert*-butoxide (1.1 g, 9.8 mmol) was first dissolved in dry THF (5 mL) and then added into the above solution. The mixture was vigorously stirred for 3 h under inert condition at room temperature. After the reaction, the crude mixture was neutralized by using acidified ethanol (200 mL, with 5% glacial acetic acid), filtered, and washed with excess ethanol. A pale yellow powder was obtained as COP-100 after being dried at 60 °C under vacuum. Yield: 0.88 g, 96%. Elemental analysis: C, 76.6%, H, 4.2%, N, 11.5%, O, 7%. COP-600 was synthesized in the same procedure using 2,2',2''-(benzene-1,3,5-triyl)triacetonitrile (0.45 g, 2.31 mmol) and 1,3,5-tris(4-formylphenyl)benzene (1.37 g, 3.5 mmol) as monomers.

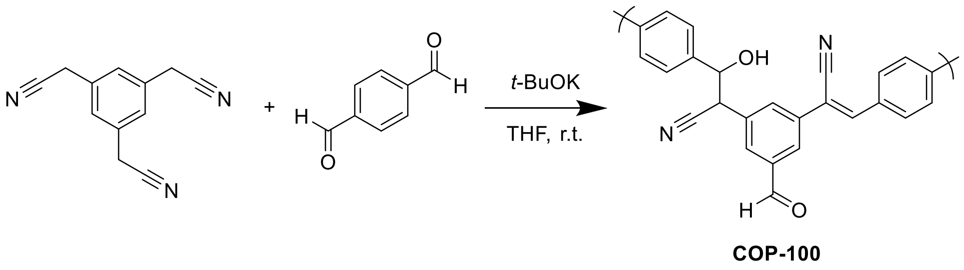

Processing of COP-100 in liquid amines

Processing of COP-100 was conducted under neat conditions with pure liquid amines. COP-100 was placed on a glass vial and the desired amount of ethylenediamine (EDA) was added on top of the COP-100. The mixture was subjected to heat treatment up to 100 °C on a hot plate to dissolve COP-100 in EDA, where a clear red polymer solution was obtained in a few seconds (<30 s). The molar ratio between COP-100

and EDA was ranged from 1:10 to 1:50, where the molar mass of COP-100 was determined on the basis of nitrogen content in COP-100 analyzed from elemental analysis. In a typical experiment, 100 mg of COP-100 was dissolved with 0.55 mL of EDA corresponding to the 10 equivalent EDA addition. The COP-100 nanoparticles were obtained by diluting the polymer solution with polar solvents such as THF and EtOH. When the polymer solution was heated further (up to 5 min), a fine polymer film formed on a substrate by the evaporation of excess EDA.

Characterization

FTIR spectrum of COP-100 was taken from KBr pellets using a Perkin-Elmer FTIR spectrometer and the rest samples were analyzed by using a Shimadzu IRTracer-100 with Gladiator 10 ATR. Elemental analysis (CHNO) was conducted on a FLASH 2000 of Thermo Scientific. Thermogravimetric analysis (TGA) was performed using a DTG-60A of Shimadzu by heating the samples to 800 °C at 10 °C min^{-1} under nitrogen atmosphere. FE-SEM (Field Emission Scanning Electron Microscopy) was carried out using a Nova 230. FE-TEM (Field Emission Transmission Electron Microscopy) was done by using a Tecnai G2 F30, 300 kV. Gravimetric CO_2 adsorption experiment of samples was recorded using a DTG-60A of Shimadzu at 40 °C under CO_2 atmosphere. The CO_2 flow rate was maintained at 50 cm^3 min^{-1} for the entire measurement using high purity CO_2 gas (99.999%).

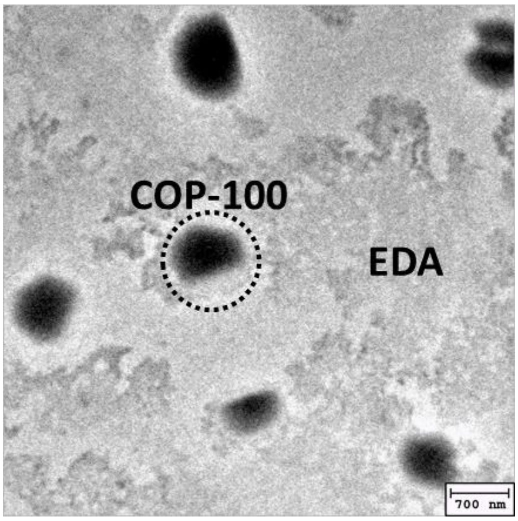

Figure S1: TEM image of COP-100 treated with EDA at 100 °C.

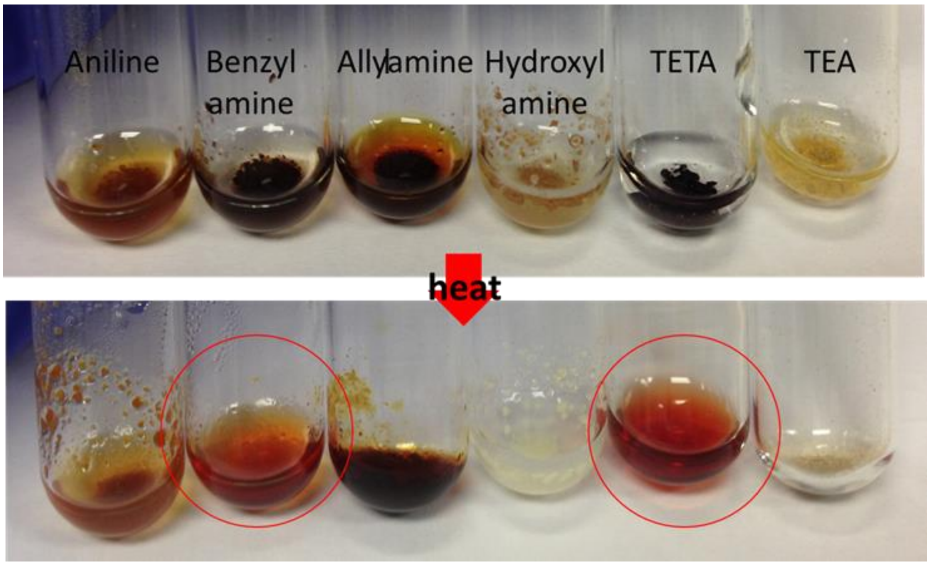

Figure S2: Variation of liquid amines for dissolving COP-100 at 100 °C. TETA: triethylenetetramine, TEA: triethylamine.

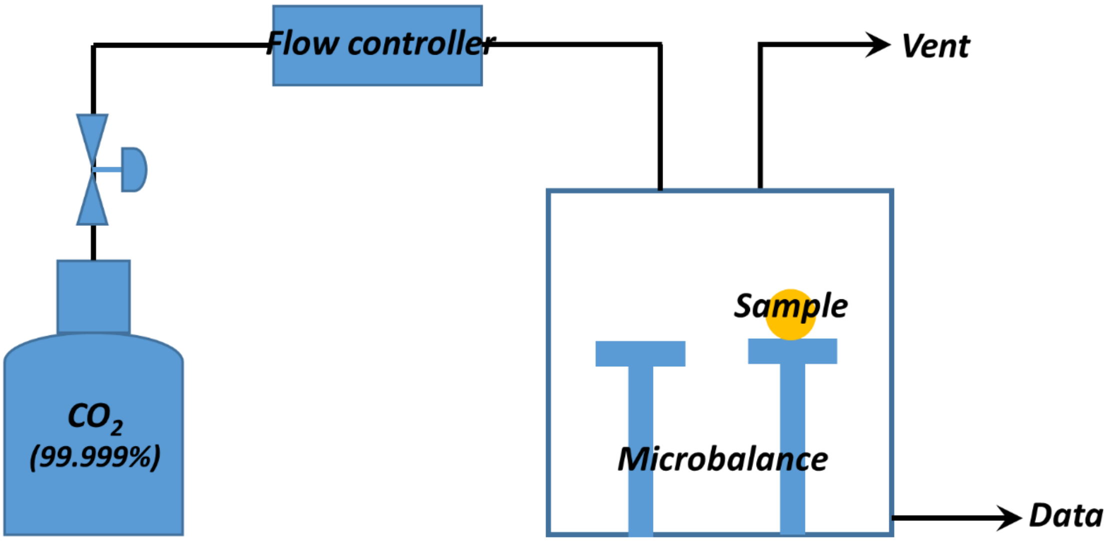

Figure S3: Schematic illustration of the experimental set-up for a gravimetric uptake of CO_2 using thermogravimetric analysis instrument.

References

1. Özdemir, E.; Thirion, D.; Yavuz, C. T. *RSC Adv.* **2015,** *5,* 69010–69015. doi:10.1039/C5RA10697D
